# Alleviating Effect of Lipid Phytochemicals in Seed Oil (*Brassica napus* L.) on Oxidative Stress Injury Induced by H_2_O_2_ in HepG2 Cells via Keap1/Nrf2/ARE Signaling Pathway

**DOI:** 10.3390/nu16172820

**Published:** 2024-08-23

**Authors:** Simin Peng, Luyan Liao, Huiqing Deng, Xudong Liu, Qian Lin, Weiguo Wu

**Affiliations:** 1 Hunan Provincial Key Laboratory of the Traditional Chinese Medicine Agricultural Biogenomics, Changsha Medical University, Changsha 410219, China; rbtpsm3773@163.com; 2 College of Food Science and Technology, Hunan Agricultural University, Changsha 410128, China; liaoluyan@126.com (L.L.);; 3 State Key Laboratory of Utilization of Woody Oil Resource, Hunan Academy of Forestry, Changsha 410018, China; 4 Institute of Bast Fiber Crops, Chinese Academy of Agricultural Sciences, Changsha 410125, China

**Keywords:** dietary phytochemicals, lipid phytochemicals, *Brassica napus* L., central composite design, oxidative stress, Keap1/Nrf2

## Abstract

α-tocopherol (α-T), β-sitosterol (β-S), canolol (CA), and sinapic acid (SA) are the four main endogenous lipid phytochemicals (LP) found in *Brassica napus* L. seed oil, which possess the bioactivity to prevent the risk of several chronic diseases via antioxidant-associated mechanisms. Discovering the enhancer effects or synergies between LP is valuable for resisting oxidative stress and improving health benefits. The objectives of this study were to identify a potentially efficacious LP combination by central composite design (CCD) and cellular antioxidant activity (CAA) and to investigate its protective effect and potential mechanisms against H_2_O_2_-induced oxidative damage in HepG2 cells. Our results indicated that the optimal concentration of LP combination was α-T 10 μM, β-S 20 μM, SA 125 μM, and CA 125 μM, respectively, and its CAA value at the optimal condition was 10.782 μmol QE/100 g. At this concentration, LP combination exerted a greater amelioration effect on H_2_O_2_-induced HepG2 cell injury than either antioxidant (tea polyphenols or magnolol) alone. LP combination could reduce the cell apoptosis rate induced by H_2_O_2_, lowered to 10.06%, and could alleviate the degree of oxidative damage to cells (ROS↓), lipids (MDA↓), proteins (PC↓), and DNA (8-OHdG↓). Additionally, LP combination enhanced the antioxidant enzyme activities (SOD, CAT, GPX, and HO-1), as well as the T-AOC, and increased the GSH level in HepG2 cells. Furthermore, LP combination markedly upregulated the expression of Nrf2 and its associated antioxidant proteins. It also increased the expression levels of Nrf2 downstream antioxidant target gene (*HO-1*, *SOD-1*, *MnSOD*, *CAT*, *GPX-1*, and *GPX-4*) and downregulated the mRNA expression levels of Keap1. The oxidative-stress-induced formation of the Keap1/Nrf2 complex in the cytoplasm was significantly blocked by LP treatment. These results indicate that LP combination protected HepG2 cells from oxidative stress through a mechanism involving the activation of the Keap1/Nrf2/ARE signaling pathways.

## 1. Introduction

Many metabolism-related chronic diseases are associated with oxidative stress [1], including obesity and its related diseases, such as diabetogenic, atherogenic, hyperglycemia, and so on [2,3]. Oxidative stress is widely occurring in biological systems and is an imbalance between reactive oxygen species (ROS) and cellular antioxidant defense systems. ROS mainly include active superoxide anions (O^2−^), hydroxyl radicals (^•^OH), and singlet oxygen (^1^O_2_) [4]. Among them, ^•^OH is the most damaging of the ROS produced via the Fenton reaction between free Fe^2+^/Cu^+^ and H_2_O_2_ [5], which can be toxic to a variety of cells like HepG2 [6] and endothelial cells [7].

As we move into the public health 4.0 era with disease prevention and health management going hand in hand, dietary phytochemicals have been explored as potential regulators of oxidative stress. Recent studies imply that dietary antioxidant application could activate the enzymatic antioxidant system or the nonenzymatic antioxidant system to scavenge ROS [8] along with activation of the Nrf2 antioxidant pathway to maintain the intracellular redox balance and protect against oxidative stress [9]. Wu et al. found that ampelopsis grossedentata (AG), an ancient medicinal and food homologous plant, rich in flavonoids, phenols, steroids, terpenoids, and so on. AG extract exhibited strong DPPH radical scavenging capacity and high oxygen radical absorbance capacity during in vitro experiments, and it may exert antioxidant effects through the activation of the Nrf2/ARE pathway [10]. Kubo et al. showed that astaxanthin, a xanthophyll carotenoid widely distributed in marine environments, increases the expression of Nrf2 and HO-1 in mice lung to ameliorate cigarette-smoke-induced emphysema [11]. In addition, the existing evidence suggests that edible oil like olive oil, an essential part of the Mediterranean diet, can be a promising intervention strategy for oxidative-stress-mediated chronic diseases, including obesity. The effect was benefits from the bioactive components in oils [12,13].

Human hepatocarcinoma HepG2 cell line is a well-differentiated transformed cell line, which is easy to culture and well characterized [14]. Using HepG2 cells, Xiao et al. [15] verified the protective effect and the relevant mechanism of cordyceps sinensis exopolysaccharide-selenium nanoparticles in ameliorating H_2_O_2_-induced injuries. Abdullah et al. [16] found the antioxidant potential and protective effect of modified sea cucumber peptides against H_2_O_2_-induced oxidative damage in vitro in HepG2 cells. It can be seen that the oxidative damage model established by HepG2 cells has been widely used in cell-based bioassays of food antioxidant activity. In this paper, therefore, the antioxidant capacities of lipid phytochemicals (LP) in *Brassica napus* L. seed oil were investigated in HepG2 cells using H_2_O_2_-induced oxidative stress.

**Figure 1:**
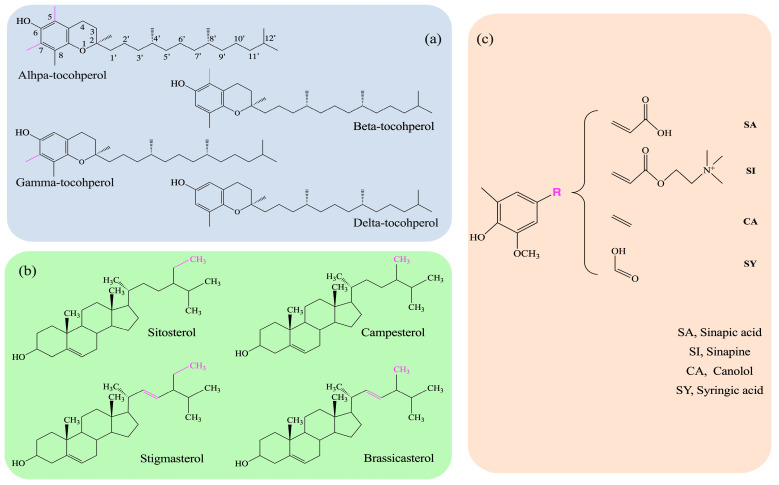
Chemical structures of the main LP in *Brassica napus* L. seed oils. (**a**) Four lipophilic isomers of tocopherols. (**b**) Four types of phytosterols. (**c**) Types of polyphenols.

LP are natural minor phytochemicals that are extracted together with oils during the extraction process. In *Brassica napus* L. seed oils, LP were mainly composed of polyphenols, tocopherols, and phytosterols (Figure 1). They possess biological activities, including suppression of cholesterol genesis, anti-inflammation, control of adipocyte function, and so on. LP are not only micronutrients, they also act as the endogenous antioxidants in edible oils. In recent years, the potential of LP in the body’s antioxidant capacity has begun to be investigated. Canolol (CA), one of the most characteristic LP of rapeseed oil, could strengthen the antioxidant defense system of HepG2 cells by upregulating the levels of GSH, SOD, and CAT, thus indicating a specific protective effect of CA on H_2_O_2_-induced oxidative stress in cells [17]. Xu et al. [18] also found that sinapic acid (SA) and CA treatment significantly reduced the oleic-acid-induced HepG2 cytotoxicity and the ROS levels and increased SOD levels. As shown in these studies, the antioxidant effect of single-lipid phytochemical has been known. However, LP exert antioxidant interactions in the body when ingested simultaneously. Depending on the ratio at which these antioxidants are combined, these antioxidant interactions may be additive, antagonistic, or synergistic [19]. Currently, few scholars have studied the lipid phytochemical mixture’s overall efficiency on antioxidant activity.

In this study, the optimal compounding concentrations of four kinds of LP (α-tocopherol (α-T), β-sitosterol (β-S), CA, and SA) to ameliorate cellular oxidative stress were investigated using a cellular antioxidant (CAA) model in combination with the central composite design (CCD) method. In addition, the protective effects and potential mechanisms of LP on the H_2_O_2_-induced oxidative damage in HepG2 cells were explored. Thereby, a thorough understanding of the potential functions of LP on oxidative stress will be revealed and, thus, provide a better natural alternative health food both for the prevention and treatment of oxidative stress injury.

## 2. Materials and Methods

### 2.1. Materials

α-T (purity > 96%), β-S (purity > 95%), and SA (purity > 98%) were purchased from Sigma Co., Ltd. (Dorset, UK). CA (purity > 98%) was obtained from Weikeqi-biotechnology Co., Ltd. (Chengdu, China). Tea polyphenols (polyphenol content > 98%, catechins > 72%, and epigallocatechin gallate > 41%), magnolol (purity > 98%), and quercetin dihydrate (QE) (purity > 97%) were purchased from Yuanye Biotechnology Co., Ltd. (Shanghai, China).

Dulbecco’s modified Eagle medium (DMEM), trypsin, 2′,7-Dichlorofluorescin diacetate (DCFH-DA), 2,2-Azobis (2-amidinopropane) dihydrochloride (ABAP), dimethyl sulfoxide (DMSO), and phosphate buffer solution (PBS) were purchased from ThermoFisher scientific Co., Ltd. (Waltham, MA, USA). Penicillin–streptomycin was obtained from Gen-view scientific Inc. (Tallmadge, OH, USA). Fetal bovine serum (FBS) and Hanks’ balanced salt solution (HBSS) were purchased from Gibco Co., Ltd. (Grand Island, NY, USA). The kits for determination of cell counting kit-8 (CCK-8), MDA, SOD, CAT, GPX, GSH, T-AOC, PC, Annexin V-FITC apoptosis, and BCA protein assays were obtained from Biyotime Biotechnology (Shanghai, China). The kits for determination of ROS, HO-1, and 8-OHdG Elisa were obtained from Jiancheng Bioengineering Institute (Nanjing, China). SYBR Premix Ex Taq was Provided by Meixuan biological technology Co., Ltd. (Shanghai, China). All other reagents were analytical-grade products from Sinopharm Chemical Reagent Co., Ltd. (Shanghai, China).

### 2.2. Cell Cultures

HepG2 cells were obtained from the laboratory of Hunan Agricultural University and were maintained and supplemented with 10% FBS and 1% penicillin–streptomycin in a humidified incubator inflated with 5% CO_2_ at 37 °C.

### 2.3. Determination of Cell Viability by Cell Counting Kit-8 (CCK-8)

The CCK-8 determination method was slightly modified according to Gong’s method [20]. HepG2 cells (5 × 10^4^ cells/well) were seeded in a 96-well culture plate with 100 μL cell culture medium in each well. After 24 h of incubation, the cells were completely adhered and the corresponding drugs (α-T, β-S, SA, CA, QE, tea polyphenols, and magnolol) were added to the experimental group with different concentrations. At the indicated time point, 10 μL CCK-8 solution was added to the corresponding wells and incubated at 37 °C with 5% CO_2_. After 2 h of cell culture, the viability of HepG2 cells was monitored using the CCK-8 assay, with the value of optical density (OD) of each well being tested by a microplate reader at 450 nm to calculate cell viability. The cell viability histogram was plotted with concentration as abscissa and cell viability as ordinate. All experiments were conducted independently in triplicate.

The concentrations of drugs used were as follows: α-T (0, 12.5, 25, 50, 100, and 200 μM), β-S (0, 25, 50, 100, 200, and 400 μM), SA (0, 31.5, 62.5, 125, 250, and 500 μM), CA (0, 5, 35, 65, 95, and 125 μM), QE (0, 1, 2, 4, 8, 16, and 32 μM), tea polyphenols (0, 3.55, 7.11, 14.22, 28.43, and 56.87 μM), and magnolol (0, 3.75, 7.51, 15.02, 30.04, and 60.07 μM). A broad range of the drugs’ concentrations was determined by references, and the final range of the drugs’ concentrations was subsequently determined by the results of pre-experiment.

### 2.4. CAA in HepG2 Cells

CAA determination followed the method described by Wolfe et al. [21]. Briefly, HepG2 cells in logarithmic growth phase were seeded in triplicates into 96-well plates (1 × 10^4^ cells/well) and cultured at 37 °C with 5% CO_2_ in the media for 24 h. After discarding the culture medium, the wells were washed with PBS (100 μL) once. Then, cells were treated for 1 h in an incubator of 37 °C and 5% CO_2_ with 100 μL of DMEM containing 25 μM DCFH-DA and different concentrations of sample mixtures. After incubation, the plate was washed twice with PBS and the medium was replaced with 100 µL of HBSS medium (containing 600 μM of ABAP). The cells were immediately placed in a microplate reader, where the excitation wavelength was set at 485 nm and emission wavelength at 538 nm. Relative fluorescence units were measured every 5 min for a period of 90 min. Each plate included triplicate control and blank wells; control wells contained cells treated with DCFH-DA and ABAP; blank wells contained cells treated with DCFH-DA and no ABAP.

After blank subtraction from the fluorescence readings, the area under the curve of fluorescence versus time was integrated to calculate the CAA value at each concentration as follows:

(1) CAA=100−(∫SA−∫BA)/(∫CA−∫BA)×100%

where ∫SA is the integrated area under the sample fluorescence versus time curve; ∫CA is the integrated area under the control fluorescence versus time curve; ∫BA is the integrated area under the blank fluorescence versus time curve; the area under each curve was calculated via integration with the Prism Data analysis software (GraphPad Prism version 7.0).

For CAA quantification, each sample was relatively quantified based on a standard curve constructed with quercetin as standard. Briefly, the CAA unit of sample was expressed in milligrams of quercetin equivalents per gram of extract.

### 2.5. Optimization of LP Combination by CCD-RSM (Response Surface Methodology)

A 4-factor CCD under RSM was applied to investigate the effect of LP on the CAA value under various combinations and to estimate the interactions effect between these parameters. Typically, a CCD contains (1) factorial points, for each variable has two values, the low and high level, (2) axial points, the extreme values of variables, and (3) center points, the medium of the values used in factorial points. In this study, α-T (A), β-S (B), SA (C), and CA (D) were the experimental factors. They were randomized and varied over five levels, with +2 and −2 as the axial points, +1 and −1 as the factorial points, and 0 as the center point, as shown in Table 1. The CCD plan is composed of 31 trials, and the selected response for the CCD model was the CAA (μmol QE/100 g). Then, the experimental results were analyzed through ANOVA to evaluate the effect of each factor and the significance of the model. The experimental design, ANOVA, and RSM plotting were carried out using Design Expert^®^ Version 12 software.

The polynomial model used for data analysis is shown as below:

(2) $$Y(CAA)=A_{0}+\sum_{i=1}^{4}A_{i}X_{i}+\sum_{i=1}^{4}A_{ii}X_{i}^{2}+\sum_{i=1}^{4}A_{ij}X_{i}X_{j}$$

where Y is the predicted dependent variable (CAA), $A_{0}$ is the intercept and $A_{i},\;A_{ii},\;A_{ij}$ represent linear, quadratic, and interaction coefficients of the model, respectively. $X_{i}$ and $X_{j}$ are the independent variables (i ≠ j).

In this part, α-T (A), β-S (B), SA (C), and CA (D) were mixed based on the design of the experiment and 31 mixed samples were obtained for testing. The ranges of these variables were designed based on CCK-8 results. Specifically, α-T (A), β-S (B), SA (C), and CA (D) stock solution were dissolved in DMEM to final concentrations of 200 μM, 500 μM, 500 μM, and 500 μM, respectively, and stored at −18 °C. The single sample was prepared by pipetting a certain amount of α-T (A), β-S (B), SA (C), and CA (D) stock solutions into a centrifuge tube, and the final volume of each sample was adjusted to a final volume of 1 mL with DMEM. All samples were mixed well and stored in a light-free plastic test tube at −20 °C until further analysis.

### 2.6. Construction and Grouping of Oxidative Stress Model

To establish the in vitro oxidative stress model, H_2_O_2_ was applied to HepG2 cells. Firstly, HepG2 cells in logarithmic growth phase were seeded in triplicates into 12-well plates (2 × 10^5^ cells/well) and cultured at 37 °C with 5% CO_2_ for 24 h. Then, the cells were separated into five treatment groups and treated with different drugs. Details as to the experimental grouping of cells were as shown in Table 2. After 24 h pretreatment, the cells were washed twice with PBS and treated with 200 μM H_2_O_2_ for 2 h. H_2_O_2_ concentration (200 μM) was determined based on the results of preliminary experiments and the required H_2_O_2_ solutions were made freshly before being used.

### 2.7. Determination of Cell Apoptosis by Annexin-V/PI

Cell apoptosis was detected using Annexin V-FITC/PI apoptosis detection kit. In brief, cell suspension was prepared from the HepG2 cell treatment in 2.6. For staining, cells were diluted to 5 × 10^6^ cells/mL in 1× binding buffer. Then, 100 μL cell suspension was taken into a flow tube and add 5 μL Annexin V-FITC was added. After gentle vortexing, the cells were incubated at room temperature for 5 min in the dark. Subsequently, 10 µL 20 µg/mL PI and 400 µL PBS were added to each tube and the samples were immediately analyzed by flow cytometry. The apoptosis measurements were repeated three times in each experiment.

The apoptosis rate was calculated according to the following formula: total apoptosis rate/(%) = early apoptosis rate/(%) + late apoptosis rate (%). Here, the left lower quadrant (Q4) represented healthy living cells, FITC−/PI−; the right lower quadrant (Q3) represented early apoptotic cells, FITC+/PI−; and the right upper quadrant (Q2) represented necrotic cells and advanced apoptotic cells, FITC+/PI+.

### 2.8. Determination of ROS, MDA, PC, and 8-OHdG in HepG2 Cells

Intracellular ROS levels were measured using the ROS assay kit. Briefly, pretreatment of HepG2 cells was according to a previous description in 2.6. Then, the cells were incubated with 10 μM DCFH-DA at 37 °C for 20 min. At the end of incubation, the cells were washed three times with serum-free medium and harvested. Finally, cell fluorescence was immediately analyzed by flow cytometry with the peak excitation wavelength for excitation at 488 nm and emission 525 nm.

The levels of MDA, PC, and 8-OHdG in HepG2 cells were all determined using test kits in accordance with corresponding instructions. Similarly, pretreatment of HepG2 cells was performed as described above. Then, the cultured cells were lysed in the cell lysis buffer for Western blotting and IP. After the completion of cell lysis, the lysate was subjected to centrifuge at 12,000 rpm for 5 min to take the supernatant. Finally, the quantifications of MDA, PC, and 8-OHdG were analyzed using a microplate reader at 532 nm, 450 nm, and 370 nm. All results were presented as the average of three independent assays.

### 2.9. Determination of T-AOC, SOD, CAT, GPX, HO-1, and GSH in HepG2 Cells

Cells were processed for oxidative stress parameters as described above in Section 2.8. For T-AOC, SOD, CAT, GPX, HO-1, and GSH analysis, they were determined by spectrophotometry according to the manufacturers’ instructions. Protein content was detected by a BCA protein assay kit. All assays were performed in at least three independent experiments and data are presented as average + SE.

### 2.10. Determination of QPCR in HepG2 Cells

The total RNA was extracted using a method reported by Chen et al. [22]. All primers (Table 3) were synthesized by Shanghai Meixuan biological science and technology Co., Ltd. (Shanghai, China). Real-time PCR was performed using the SYBR Premix Ex Taq kit and the ABI 7500 real-time PCR system, with β-actin as an internal reference. The dissociation curves for each reaction were checked and the relative expression levels of each target gene were calculated using the 2^−ΔΔct^ method (ΔCt = Ct (Target gene) − Ct (Actin), ΔΔCt = ΔCt (Treatment) − ΔCt (Control)) [23]. Each reaction was performed with at least 3 biological replicates.

### 2.11. Determination of Nrf2 and Keap1 Expression by Western Blotting Analysis

Western blot analysis followed the method described by Zhang et al. [24], total proteins from HepG2 cells treated in 2.6 were extracted by using RIPA lysis buffer, and the protein concentration was determined according to the manufacturer’s instructions using BCA Protein Assay Kit. Samples were separated on 12% SDS-PAGE gels before being transferred to PVDF membranes. After the membranes were blocked with 5% BSA, they were incubated with primary antibodies overnight at 4 °C. The detected proteins included Keap1 and Nrf2, and GAPDH was used as the internal reference. Afterwards, the membrane was washed (3 × 5 min) in TBST before incubation in the secondary antibody solution in TBST containing HRP-conjugated anti-rabbit secondary antibody (1:5000) for 2 h at room temperature. The membrane was then washed again and incubated for 2 min in enhanced chemiluminescence reagent and visualized using a gel imaging system. Band volumes were quantified using ImageJ (NIH). The value for each protein was normalized by its corresponding GAPDH level.

### 2.12. Statistical Analysis

Data were statistically analyzed using SPSS statistical program (version 17.0; IBM Corp., Armonk, NY, USA). Results were represented as the mean ± standard deviation. One-way ANOVA was used to compare across groups or conditions, and t-tests were used to compare two groups. Means with differently lettered superscripts differ significantly at the probability of *p* value < 0.05.

## 3. Results

### 3.1. Effects of LP, Tea Polyphenols, and Magnolol on the Cell Activity of HepG2 Cells

**Figure 2:**
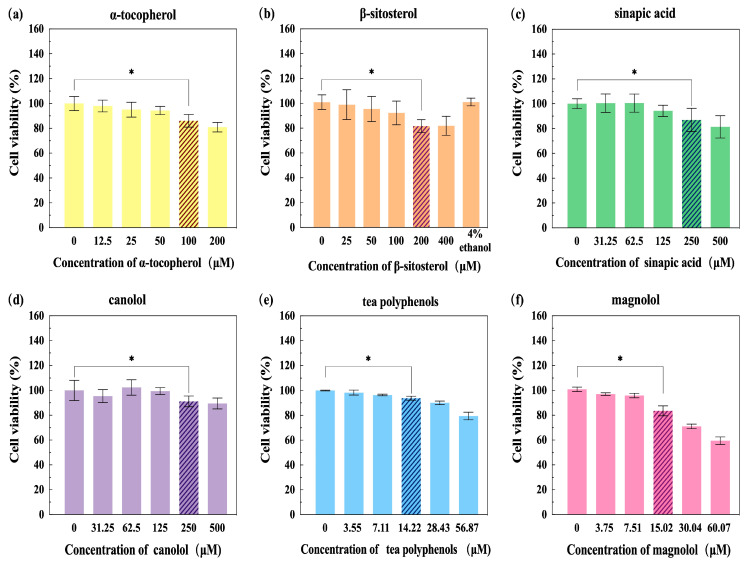
Cell viability of HepG2 cells after incubation with α-tocopherol (**a**), β-sitosterol (**b**), sinapic acid (**c**), canolol (**d**), tea polyphenols (**e**), and magnolol (**f**) for 24 h detected by CCK-8 assay. * *p* < 0.05 versus 0 concentration group.

To investigate the cytotoxicity of LP (α-T, β-S, SA, and CA) on cultured HepG2 cells, CCK-8 assays were performed to determine the cell viability; the results are shown in Figure 2a–d. For α-T (Figure 2a) and β-S (Figure 2b), cell viability decreased with increasing concentration of α-T and β-S before statistically significantly reducing at an α-T concentration of 100 μM and β-S concentration of 200 μM, respectively (*p* < 0.05). For SA (Figure 2c) and CA (Figure 2d), statistically significantly reductions were observed when the concentrations were less than 250 μM (*p* < 0.05). Hence, 50 μM of α-T, 100 μM of β-S, 125 μM of SA, and 125 μM of CA were selected as the safe and effective concentrations for the subsequent experiments.

Subsequently, the effects of different concentrations of tea polyphenols and magnolol on the activity of HepG2 cells were systemically compared. As shown in Figure 2e and Figure 2f, there was a significant difference between cell activity at a tea polyphenol concentration of 14.22 μM and magnolol concentration of 15.02 μM, respectively (*p* < 0.05). Therefore, 7.11 μM of tea polyphenols and 7.51 μM of magnolol were regarded as the highest safe concentrations for the subsequent experiments.

### 3.2. CAA Standard Curve

**Figure 3:**
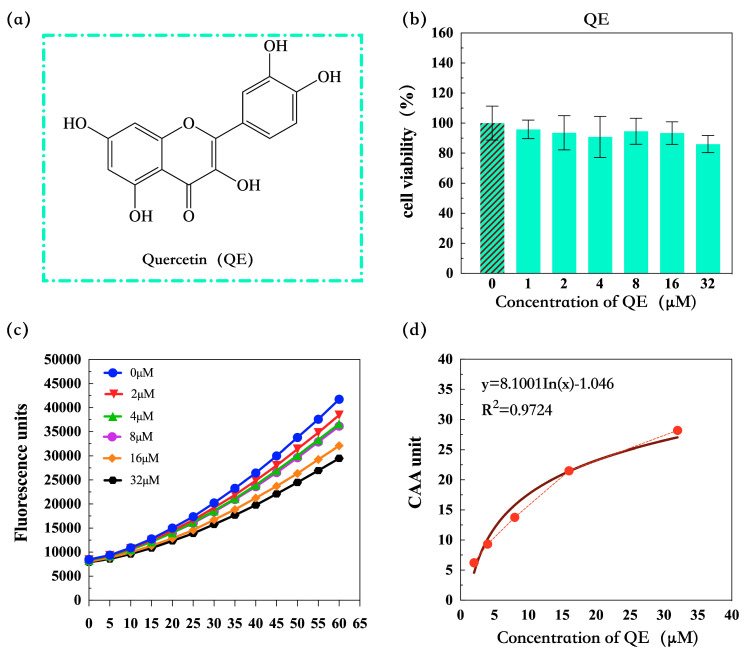
Cell viability of HepG2 cells after incubation with QE for 24 h detected by CCK-8 assay; (**a**) chemical structure of QE. (**b**) Cell viability of HepG2 cells after incubation with QE. (**c**) Peroxyl radical-induced oxidation of DCFH to DCF in HepG2 cells and inhibition of oxidation by QE (mean ± SD, n = 3). (**d**) The CAA standard curve of QE in the concentrations of 0, 2, 4, 8, 16, and 32 μM.

To determine whether there is a cytotoxic effect of QE to HepG2 cells, a CCK-8 assay was firstly performed (Figure 3b) and the result showed that QE had no effect on HepG2 cell activity at concentrations up to 32 μM, so time–fluorescence curves of different QE concentrations (0, 2, 4, 8, 16, and 32 μM) were drawn (Figure 3c).

As shown in Figure 3c, a time–response curve was independently obtained for each concentration of QE at 13 different time points. Although they reflect the same trend for each concentration, we observed a decreasing trend in fluorescence value with increasing QE concentration. Then, the CAA values of different QE concentrations were calculated according to the formula listed in 2.4 and the CAA unit was plotted versus the QE concentration. Finally, a nonlinear regression was performed (Figure 3d) and revealed that the equation of the CAA standard curve is y = 8.1001 In(x) − 1.046 and the regression coefficient is R^2^ = 0.9724.

### 3.3. Optimization of LP Concentrations by Central Composite Design (CCD)

#### 3.3.1. CCD Analysis and Model Fitting

Table 4 shows the design matrix of the variables and the corresponding experimental results. High values of CAA were obtained when high concentrations of α-T (A) and low concentrations of SA (C) and CA (D) were used. Meanwhile, by increasing the concentration of CA (D), the value of CAA will be increased as well. The significance and magnitude of the effects of the main variables and all possible interactions (linear, two-factor interaction (2FI), quadratic, or cubic) on the response variables were determined using ANOVA (Table 5).

The sum of squares analysis of the sequential model revealed that linear and quadratic functions were significant (<0.0001). The experimental data were better matched to the quadratic function than the linear function, as seen by its lower mean square value (0.44). In comparison to other functions, the lack of fit tests revealed that the quadratic function was insignificant under the set conditions, with low F-value (1.08) and the highest *p*-value (0.4819). Furthermore, the mean square of the residual error of a quadratic function (0.077) was in accord with the model’s pure error (0.071), indicating that the quadratic model could accurately anticipate the results. Despite having the greatest R^2^ (0.9795), the cubic function had a high PRESS (28.76) indicating that the cubic model’s data fitting and new data interpretation did not perform well. Thus, the quadratic model, with the lowest PRESS (5.02), as well as the greatest anticipated R^2^ (0.926), accurately captured the results of the model fitting based on the 31 experimental runs.

ANOVA evaluations for quadratic modeling of CAA are shown in Table 5. The model obtained had a very low *p*-value (<0.001) and the lack of fit *p*-value of 0.4819 (>0.05) implied that the lack of fit was not significant relative to the pure error. Meanwhile, the adequate precision of 5.02 (>4) indicates that the signal is adequate. Thus, this model may accurately characterize the response behavior based on the independent variables. According to ANOVA, α-T, β-S, CA, α-T/SA, α-T/CA, β-S/SA, β-S/CA, SA/CA, and CA^2^ were the significant model terms because of their *p*-values with the criterion <0.05. Furthermore, the terms SA, α-T/β-S, α-T^2^, β-S ^2^, and SA^2^ were insignificant by their *p*-values of 0.7574, 0.0726, 0.8399, 0.3818, and 0.3078, respectively. As the quadratic model was well fitted with the data and predicted accurately the new data, the CAA response function of the four variables can be described as follows:

Y(CAA) = 4.38 + 0.16A − 0.29B + 0.018C + 0.65D − 0.13AB − 0.36AC − 0.34AD − 0.37BC − 0.61BD + 0.37CD − 0.011A^2^ − 0.046B^2^ − 0.054C^2^ − 0.24D^2^ (3)

where A—single effect of α-T concentration, B—single effect of β-S concentration, C—single effect of SA concentration, D—single effect of CA concentration, AB—interactive effect between factors A and B, AC—interactive effect between factors A and C, AD—interactive effect between factors A and D, BC—interactive effect between factors B and C, BD—interactive effect between factors B and D, CD—interactive effect between factors C and D, A^2^—quadratic effect of factor A, B^2^—quadratic effect of factor B, C^2^—quadratic effect of factor C, and D^2^—quadratic effect of factor D.

#### 3.3.2. Response Surface (RSM) Analysis

**Figure 4:**
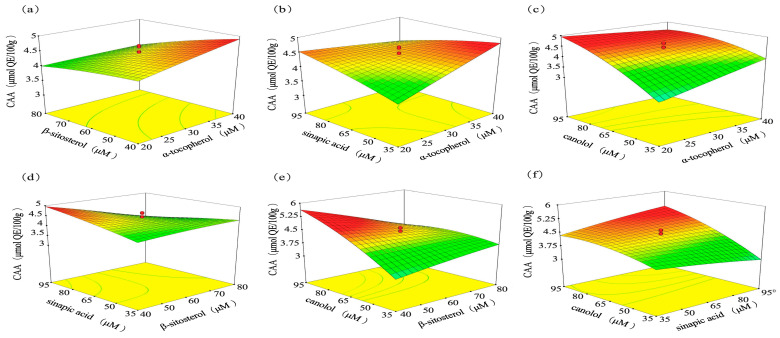
Three-dimensional surface plots from CCD model representing the effects of LP concentration on CAA value. The interactions between (**a**) α-T and β-S, (**b**) α-T and SA, (**c**) α-T and CA, (**d**) SA and β-S, (**e**) CA and β-S, and (**f**) CA and SA were analyzed.

Figure 4 shows the corresponding response surface plots for the CAA involving two experimental factors, respectively. Take Figure 4a as an example, it shows the interaction of α-T concentration and β-S concentration on CAA value. By comparing the surface inclination of Figure 4a–f, we found that, for the CAA value, the most noticeable response surface curvatures were produced by the tested ranges of β-S (B) and CA (D). Among them, the rise in CA concentration mainly brought about a rise in CAA value, as evidenced by the highest F value (Table 5) of this factor. As shown in Figure 4e, the value of CAA raised gradually with increasing CA concentration, and the maximum CAA obtained was nearly 5.5 μmol QE/100 g. Additionally, CAA was also influenced significantly by the interaction of α-T * SA/CA, β-S * SA/CA, and SA * CA (*p* < 0.05) (Table 5).

#### 3.3.3. Optimization and Validation of the Regression Model

To obtain the optimal LP combination that allows for the maximum CAA value, numerical optimization was used in RSM. In this case, CAA at the maximum level and α-T/β-S/SA/CA concentration in the range between −2 and 2 (coded level) were set for maximum desirability. The calculation result showed that LP combination using α-T = 10 μM, β-S = 20 μM, SA = 125 μM, and CA = 125 μM provided the optimal maximum CAA value. Meanwhile, validation experiments were performed in triplicate for the optimum combination and CAA was determined to be 10.782 μmol QE/100 g. This value was close to the theoretical value predicted by Equation (3), that is CAA_pred_ = 12.263 μmol QE/100 g (desirability = 0.938). This indicated that the RSM approach showed satisfactory correlation between predicted and experimental values. Thereby, the model obtained by CCD-RSM was effective and reliable for predicting the optimal antioxidant effects of LP combination, and these optimal conditions obtained could be recommended to be applied to the follow-up research.

### 3.4. Effect of LP, Tea Polyphenols, and Magnolol on HepG2 Cell Apoptosis

**Figure 5:**
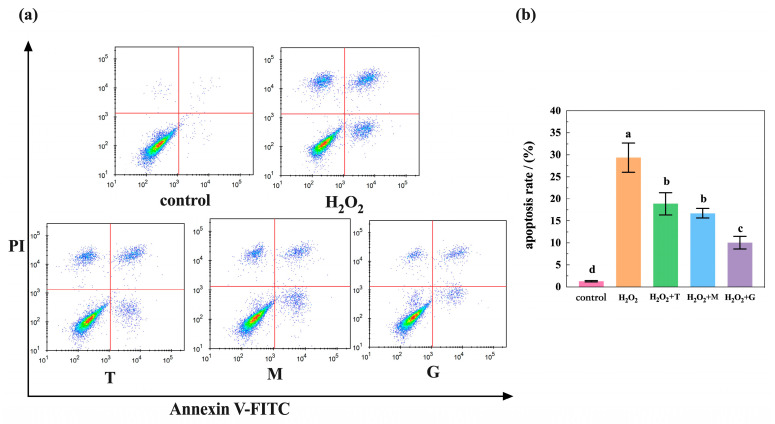
LP (G), tea polyphenols (T), and magnolol (M) alleviated H_2_O_2_-induced cell apoptosis. (**a**) Flow cytometry data of HepG2 cells at different conditions. Control group, control without antioxidants and H_2_O_2_; H_2_O_2_ group_,_ H_2_O_2_-induced injury model with only addition of 200 μM H_2_O_2_; T group, tea polyphenols treatment (addition of tea polyphenols + H_2_O_2_); M group, magnolol treatment (addition of magnolol + H_2_O_2_); G group, LP treatment (addition of LP combination + H_2_O_2_). (**b**) Statistical results of HepG2 cell apoptosis rate. Data expressed as mean ± SE (n = 3); the different lowercase letters represent statistical difference between the groups. Statistical markers a/b/c/d (*p* < 0.05).

To analyze the apoptosis of HepG2 cells treated with H_2_O_2_ and various antioxidants (LP, tea polyphenols, and magnolol), the cells were detected using Annexin V-FITC/PI staining. As exposed in Figure 5a, these scatter plots indicate that cells in the Q1, Q2, Q3, and Q4 quadrants were necrotic, late apoptotic, early apoptotic, and live cells, respectively. Corresponding histograms showing quantification of apoptotic cell rates are shown to the right (Figure 5b). In the control group, there was an insignificant number of cells in the early and late apoptotic stages with 98.71% of live cells. When HepG2 cells were exposed to 200 μM H_2_O_2_ alone, a considerable number of cells (29.38%) were in the apoptosis phase. However, a further decrease in cells treated with tea polyphenols, magnolol, and LP before induction with H_2_O_2_ showed a significant decrease (*p* < 0.05) in the apoptotic cell rates of 18.89%, 16.73% and 10.06%, respectively. This result indicates that antioxidant pretreatment inhibited H_2_O_2_-induced apoptosis. Among them, LP testing could obtain the best protection effect for cells.

### 3.5. Effect of LP, Tea Polyphenols and Magnolol on H_2_O_2_-Induced Oxidative Stress Damage in HepG2 Cells

**Figure 6:**
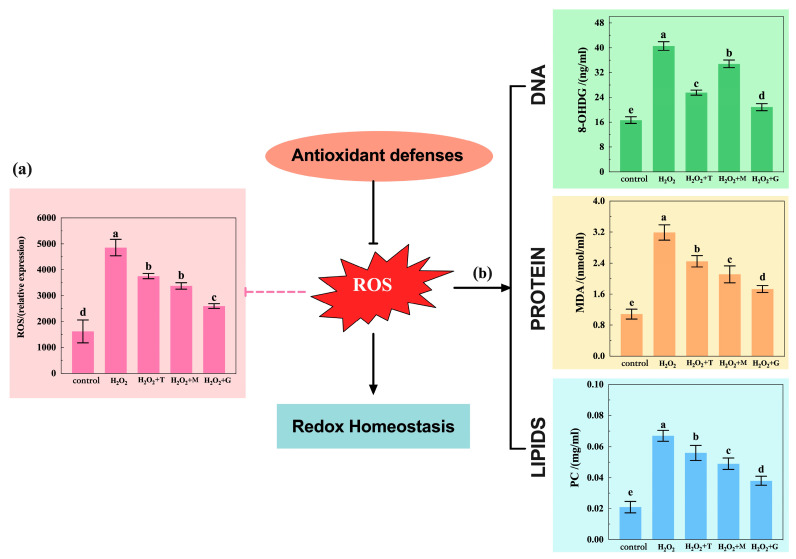
LP (G), tea polyphenols (T), and magnolol (M) alleviated the oxidative stress damage in H_2_O_2_-mediated HepG2 cells. (**a**) ROS levels were measured using DCFH-DA staining via flow cytometry; (**b**) detection of the effect of oxidative damage on DNA (8-OHdG level), protein (MDA level), and lipid (PC level). The control group was not subjected to any treatment. The H_2_O_2_ group was only treated with 200 μM H_2_O_2_ for 2 h. For the other three drug groups, the HepG2 cells were treated with G, T, or M for 24 h and then incubated for 2 h with 200 μM H_2_O_2_. All the level intensities were quantified, and results are expressed as mean ± SEM. The letters a, b, c, d, and e in the figures represent significant differences between different letters (*p* < 0.05). (T: abbreviation of tea polyphenols; M: abbreviation of magnolol; G: abbreviation of group of LP.)

From the results shown in Figure 6, the ROS, MDA, PC, and 8-OHdG content significantly increased in the H_2_O_2_ group (*p* < 0.05) when compared with those of the control group. However, the LP, tea polyphenols, and magnolol group reversed the H_2_O_2_-induced increase in ROS, MDA, PC, and 8-OHdG levels. The ROS content (Figure 6a) was gradually decreased in the following order: tea polyphenols group> magnolol group > LP group and similar trends were also seen in the change in MDA and PC content (Figure 6b). For 8-OHdG level, the magnolol group was significantly higher than the tea polyphenols group and the tea polyphenols group was significantly higher than the LP group (*p* < 0.05). Overall, the decreasing effect was most prominent in the group treated with LP.

### 3.6. Evaluation of LP, Tea Polyphenols, and Magnolol on Oxidative Stress in HepG2 Cells

**Figure 7:**
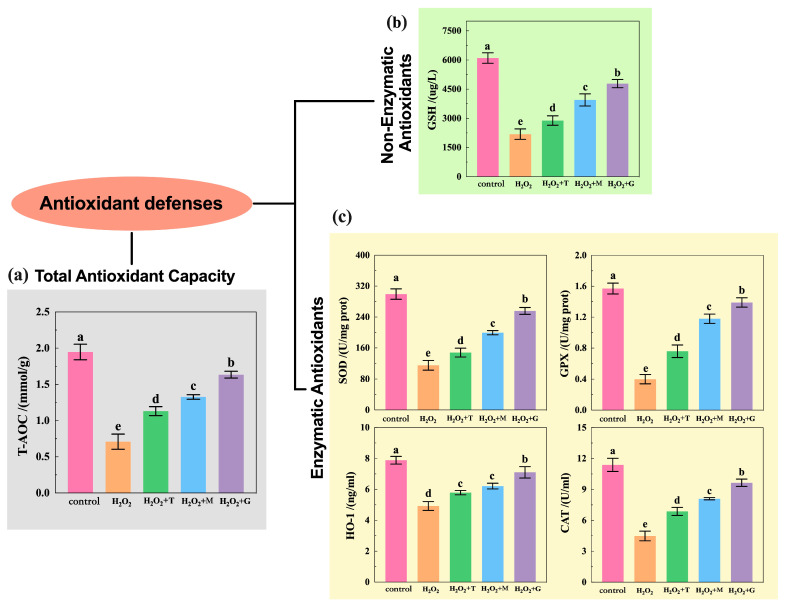
Activity of (**a**) T-AOC, (**b**) GSH, and (**c**) antioxidant enzymes (SOD, GPX, HO-1, and CAT) in HepG2 cells. The control group was not subjected to any treatment. The H_2_O_2_ group was only treated with 200 μM H_2_O_2_. H_2_O_2_ + T group, tea polyphenols treatment (addition of tea polyphenols + H_2_O_2_); H_2_O_2_ + M group, magnolol treatment (addition of magnolol + H_2_O_2_); H_2_O_2_ + G group, LP treatment (addition of LP combination + H_2_O_2_). Data expressed as mean ± SE (n = 3); the different lowercase letters represent statistical difference between the groups. Statistical markers a/b/c/d/e (*p* < 0.05).

Total antioxidant capacity (T-AOC) and measurements of nonenzymatic and enzymatic antioxidant levels are frequent techniques for estimating oxidative stress. Of these, GSH is one of the major nonenzymatic tissue antioxidants. SOD, GPX, CAT, and HO-1 constitute the main components of the antioxidant enzyme system. Compared with untreated control, T-AOC level (Figure 7a), GSH level (Figure 7b), SOD, GPX, HO-1, and CAT levels (Figure 7c) were all significantly decreased in the H_2_O_2_ alone treatment (63.55%, T-AOC; 64.16%, GSH; 61.41%, SOD; 74.52%, GPX; 37.49%, HO-1; and 60.54%, CAT). In contrast, SOD, GPX, CAT, HO-1, GSH, and T-AOC displayed increased activities in all the groups after antioxidant treatment as compared to the H_2_O_2_ group, and the increase was more significant in LP groups than in the others.

### 3.7. LP, Tea Polyphenols, and Magnolol Modulate Relative Gene Expression Levels in HepG2 Cells

**Figure 8:**
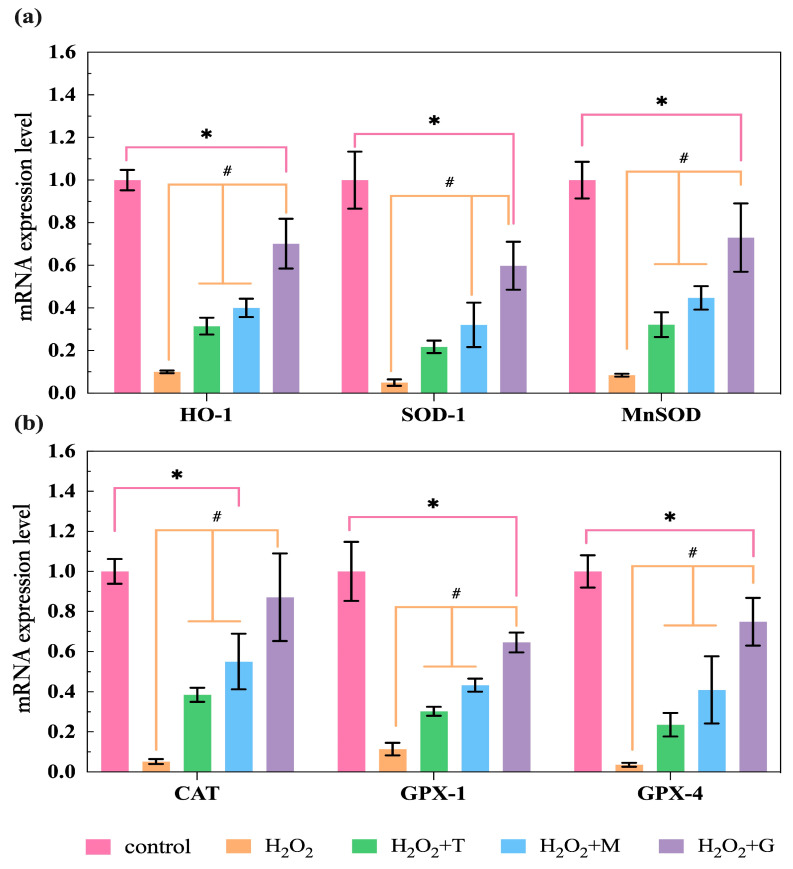
LP(G)/tea polyphenols(T)/magnolol(M)-mediated gene expression changes in HepG2 cells. The expression of (**a**) *HO-1*, *SOD-1*, and *MnSOD* and (**b**) *CAT, GPX-1*, and *GPX-4* were determined by measuring the mRNA levels with qPCR. All results are expressed as mean ± SEM. * *p* < 0.05, versus control; # *p* < 0.05, versus H_2_O_2_ group. The control group was not subjected to any treatment. The H_2_O_2_ group was only treated with 200 μM H_2_O_2_. H_2_O_2_ + T group, tea polyphenols treatment (addition of tea polyphenols + H_2_O_2_); H_2_O_2_ + M group, magnolol treatment (addition of magnolol + H_2_O_2_); H_2_O_2_ + G group, LP treatment (addition of LP combination + H_2_O_2_).

To further determine whether LP, tea polyphenols, and magnolol affected the expression of oxidative-stress-related genes in HepG2 cells, relative gene expression levels of antioxidant enzymes (HO-1, SOD-1, MnSOD, CAT, GPX-1, and GPX-4) were measured by qPCR. The results are presented in Figure 8. The relative gene expression levels of HO-1, SOD-1, MnSOD, CAT, GPX-1, and GPX-4 were all significantly decreased in the H_2_O_2_ alone treatment (89.98%, HO-1; 95.02%, SOD-1; 91.56%, MnSOD; 94.84%, CAT; 88.58%, GPX-1; and 96.44%, GPX-4) when compared with untreated control. However, it is gratifying that the HO-1, SOD-1, MnSOD, CAT, GPX-1, and GPX-4 expression levels displayed increased activities after treatment with LP, tea polyphenols, and magnolol as compared to the H_2_O_2_ group, and the increase was more significant in LP groups than in the others.

### 3.8. LP, Tea Polyphenols, and Magnolol Activate Nrf2/Keap1 Pathway and Upregulate the Expression of Nrf2

**Figure 9:**
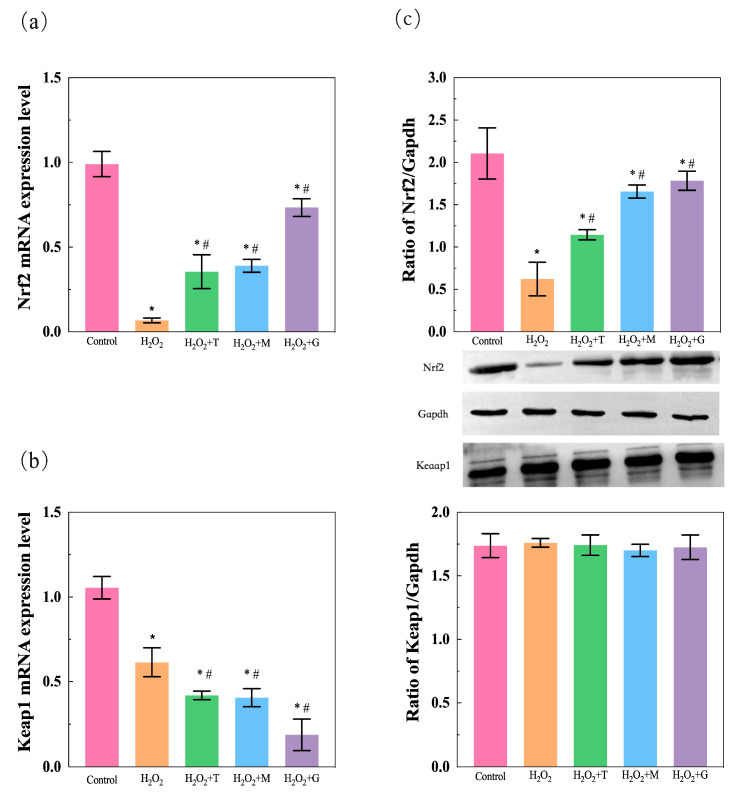
LP (G)/tea polyphenols (T)/magnolol (M)-mediated Nrf2/Keap1 gene and protein expression changes. The expression of (**a**) *Nrf2* and (**b**) *keap1* was determined by measuring the mRNA levels with qPCR. (**c**) Nrf2/Keap1 expression was determined by measuring the protein levels with WB. All results are expressed as mean ± SEM. * *p* < 0.05, versus control; # *p* < 0.05, versus H_2_O_2_ group. The control group was not subjected to any treatment. The H_2_O_2_ group was only treated with 200 μM H_2_O_2_. H_2_O_2_ + T group, tea polyphenols treatment (addition of tea polyphenols + H_2_O_2_); H_2_O_2_ + M group, magnolol treatment (addition of magnolol + H_2_O_2_); H_2_O_2_ + G group, LP treatment (addition of LP combination + H_2_O_2_).

Results in Figure 9 reflected the effect of LP, tea polyphenols, and magnolol on Nrf2/Keap1 pathway within H_2_O_2_-mediated HepG2 cell injury through qPCR and WB assays. Exposure of the HepG2 cells to H_2_O_2_ (200 μM) for 2 h led to significant decreases in the mRNA expressions of Nrf2 (Figure 9a) and Keap1 (Figure 9b). These effects for Nrf2 mRNA expression level were reversed by treatment with tea polyphenols, magnolol, and LP, while keap1 mRNA expression levels continue to fall despite of the same treatment as Nrf2.

Bar graphs and photos summarizing the WB results are shown on the right. As shown in Figure 9c, the relative protein expression of Nrf2 was significantly downregulated in H_2_O_2_-mediated HepG2 cells when compared to the control group, whereas the expressions of these proteins were markedly upregulated by tea polyphenols, magnolol, and LP pretreatment under oxidative stress. Of note, despite these HepG2 cells also being exposed to H_2_O_2_ treatment and pretreatment with tea polyphenols, magnolol, and LP, no significant change in the relative protein expression of Keap1 was observed.

## 4. Discussion

In this study, the antioxidant effects of α-T, β-S, CA, and SA were evaluated using the CCD-RSM method combined with CAA assay for the first time. The combination of RSM and CCD as a statistical and mathematical approach can simultaneously optimize multiple parameters in responses, enabling affordable, feasible, and comprehensive optimization [25]. The method has been successfully applied to formulation optimization of edible coating, drug carrier, and so on [26,27]. CCD-RSM is useful in identifying the four factors’ (α-T, β-S, CA, and SA) interactions, which were considered as independent variables, and various LP combinations were investigated to achieve optimal antioxidant capacity (response). CAA was an important tool for screening substances with potential biological antioxidant activity. This method has been commonly used with HepG2 cells to quantify the antioxidant activities of extracts and dietary supplements in food, fruit, and vegetables under physiological conditions [28,29]. Recently, this method was also applied to edible oils and their endogenous substances. Liu et al. [30] measured the antioxidant abilities of 15 kinds of edible oils using CAA methods and identified the characteristic phenols in flaxseed oil, sesame oil, and olive oil that play a vital role in cell antioxidant activity. So, CAA was used as an indicator to explore responses for different LP combinations. Finally, the results of RSM (Figure 4) and Equation (3) showed consistency behavior. CA was found to be the most significant factor that impacts CAA value; this result was in accordance with the findings of Wakamatsu D et al. [31]. In their study, CA is one of the most effective active ingredients in the elimination of free radical (ROO·), and the effect proved even greater than other common antioxidants, including α-T, β-carotene, and QE and so on. Similarly, Natalia et al. [32] found that CA and SA can be deployed as antioxidants in linoleic-acid-rich oils but canolol elicits a stronger protective effect (over fourfold).

Evidence suggests combinations of antioxidants could be more effective than single antioxidant alone because, when antioxidants are combined, they decrease vulnerability to other agents or influence each other synergistically or interact with other physiological antioxidants. As shown in Table 5, for the interaction effect, AC, AD, BC, BD, and CD are significant for CAA, which means that LPs (except for α-T/β-S) have a significant effect on the CAA, indicating that the CAA level is significantly affected by LP contents and combination and results in different levels of antioxidant capacity. Among them, the β-S and CA interaction has the greatest impact on the CAA value, according to coefficients in Equation (3). In China, the research on LP started relatively late, and the corresponding investigations were limited. To date, Liu et al. [19] investigated the antioxidant interaction of α-tocopherol, γ-oryzanol, and phytosterol in rice bran oil; they found the inhibition of phytosterol on α-tocopherol and the formation of hydrogen bond between γ-oryzanol and phytosterol. Our research is one of the few investigations assessing antioxidant interaction of LP.

For LP, many reports indicate that it possesses powerful antioxidant properties and plays an important role in many effects like lowering of blood lipids, prevention of adipocyte synthesis, and reduction in inflammatory response. Although the protective effect of LP has been reported, very little is known about its antioxidant activity and underlying mechanism in relation to H_2_O_2_-induced apoptosis. H_2_O_2_ is one of the most abundant ROS and is both highly reactive and toxic; it can cause serious damage to cells by attacking biomolecule membranes and eventually lead to apoptosis. It has been utilized extensively as a model exogenous oxidative-stress-mediated experiment in cells. In our experiments, when HepG2 cells were exposed to H_2_O_2_, the apoptosis rate of HepG2 cells was markedly increased (Figure 5). And, with ROS production being significantly accumulated, attack occurred in DNA, protein, and lipids regions and the levels of ROS 8-0HdG, PC, and MDA in Figure 6 were strongly upregulated, reflecting the extent of damage to the HepG2 cell system caused by oxidative stress impacts. This is consistent with several previous studies reported on red blood cells and HEK293 cells [33,34].

**Figure 10:**
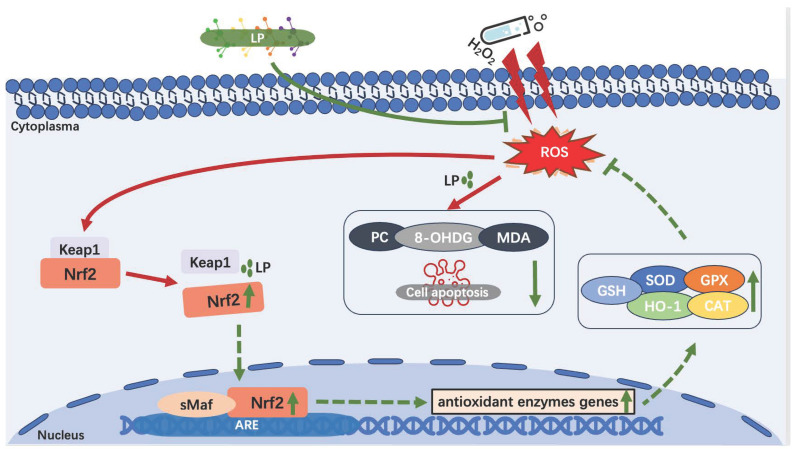
Schematic overview of the putative antioxidant mechanisms of action of LP inside HepG2 cells. Red solid arrows represent H_2_O_2_ stimulation induces oxidative stress; Green dotted arrows represent activation of Nrf2/ARE pathway, the arrow represents activation while the flat arrow represents inhibition; Green solid flat arrows represent ROS inhibition by LP; Upward and downward green solid arrows represent increase and decrease, respectively.

This study focusses on antioxidant effects of LP. The efficacy compared either of two commonly used natural antioxidants, namely tea polyphenols or magnolol. Tea polyphenols, natural plant flavonoids present in the tea, possess the bioactivity to influence the pathogenesis of chronic diseases associated with antioxidant defense mechanisms [35] and are widely used as an antioxidant for fried pasta products, meat products, and edible oils. Magnolol, bi-phenol compounds present in the magnoliae officinalis, shows antioxidant, metabolic regulation, antibacterial, and anti-inflammatory effects [36] and has been shown to be effective in improving body health and production performance of poultry and also exhibits some antioxidant effects on oils and fats. Tests showed that the pretreatment of tea polyphenols, magnolol, and LP combination effectively reduces H_2_O_2_-induced apoptosis and ROS, 8-0HdG, PC, and MDA levels (Figure 6), and LP combination achieved the best performance. These results suggested that the three antioxidants (especially LP) may reduce apoptosis and reduce the extent of damage to the HepG2 cell system via inhibiting the excessive production of ROS (Figure 10).

The Nrf2/ARE signaling pathway is an important endogenous defense system in the body and has been instrumental for many important discoveries in the field of antioxidants [37]. Nrf2 is a transcription factor that activates the expression of genes of antioxidant enzymes, including HO-1, SOD-1, MnSOD, CAT, GPX-1, GPX-4, and so on. Keap1, the cytosolic protein which prevents Nrf2 translocation to the nucleus, is considered to be the receptor of oxidative stress. As shown in Figure 10, under normal or basal conditions, Nrf2 interacts with Keap1 in the cytoplasm and relies on the ubiquitination reaction of 26S proteasome to maintain a low-content inactive and stable state [9]. During oxidative stress, Keap1 can be oxidized by ROS, resulting in conformational changes that release Nrf2 [38], and the interaction of Nrf2/Keap1 can also be altered by phosphorylation of Nrf2 [39]. The activated Nrf2 enters the nucleus and could form dimers with sMaf protein, activating the transcription expression of antioxidant enzymes genes. Then, it allows the expression of antioxidant enzymes and is used to eliminate oxidative stress. Figure 8 and Figure 9 showed that LP used in this study significantly increased the relative expression level of different antioxidant enzymes genes (*HO-1*, *SOD-1*, *MnSOD*, *CAT*, *GPX-1*, and *GPX-4*), with the increase in the genes and protein levels of Nrf2 and decrease in the relative gene expression level of Keap1. In addition, LP also markedly increased the activities of HO-1, CAT, SOD, and GSH-Px (Figure 7). In cells, enzyme-catalyzed antioxidant system and nonenzymatic system are the two defense ways to protect the body from oxidative stress; they decrease the ROS production, detoxify ROS, and stimulate recovery from ROS-induced damages [40]. For enzyme-catalyzed antioxidant systems (e.g., HO-1, CAT, SOD, GSH-Px, and so on), they work conjointly to maintain the intracellular redox balance. SOD catalyzes the dismutation of superoxide radical into O_2_ or H_2_O_2_, and the latter is further decomposed to H_2_O and O_2_ by CAT [41]. GSH-PX prevents the formation of toxic hydroxyl and peroxyl radicals via providing electrons to H_2_O_2_ and lipid peroxides [42]. For nonenzymatic systems (e.g., GSH), it is a tri-peptide consisting of cysteine, glutamate, and glycine; the reactive thiol group of the cysteine residue (-SH) provides the reducing power. GSH is converted to oxidized glutathione (GSSG) under oxidative stress and then recycled into the reduced state by GSH-reductase (GSR) [43]. Our results indicated that LP may improve the antioxidant ability via enhancing the antioxidant enzyme capacity. So far, CA [44], α-T [45], β-S [46], and SA [47] have been proven adequate to be used alone for relieving oxidative stress by activating or assisting activation of Nrf2 in cellular or animal models. Li et al. [48] found that tea polyphenols could protect RAW264.7 cells against H_2_O_2_-induced oxidative injury by increasing the levels of antioxidant enzymes and the expression of Nrf2 and HO-1. Liu et al. [49] reported that magnolol can upregulate the expression of Nrf2 and HO-1 and increase the antioxidant enzymes of GSH-Px and SOD levels, thereby attenuating alcohol-induced pathologic damage. Our results are clearly consistent with these experimental observations. In a word, these results indicate that the beneficial effects of LP appear to involve the activation of Nrf2/ARE signaling pathways (Figure 10) and enhance the activity of antioxidant enzymes.

Our work unequivocally indicated the interaction between α-T, β-S, CA, and SA and demonstrated that the combination of α-T, β-S, CA, and SA exerted an antioxidant effect in H_2_O_2_-induced HepG2 cells. This is the first study, to the best of our knowledge, to demonstrate the antioxidant function and mechanism of the combination of α-T, β-S, CA, and SA. And the combination of α-T, β-S, CA, and SA exerted significant effects compared to the effects of tea polyphenols and magnolol, which also illustrate the exerted synergistic effects in the LP combination to reduce oxidation damage. However, we acknowledge certain limitations of our study, including the use of a single liver cancer cell line, only conducting in vivo experiments, and lacking in vitro functional experiments. Further studies involving other different cell lines and in vivo experiments in animal models or human clinical trials are required to completely elucidate the molecular mechanism of how these LP prevent damages from ROS.

## 5. Conclusions

Currently, natural antioxidants from plants have attracted more attention due to the huge benefit of nutrition and safety. In the present study, an integrative strategy based on the CAA biological method coupled with CCD-RSM data analysis platform was applied for the first time to reveal the antioxidant activities of four kinds of LP in *Brassica* *napus* L. seed oil comprehensively.

The experimental results showed that the optimal LP combination (α-T = 10 μM, β-S = 20 μM, SA = 125 μM, and CA = 125 μM) was selected from all 31 combinations to maximize the efficiency of antioxidant effects mediated in *Brassica napus* L. seed oil. Furthermore, the LP combination of *Brassica napus* L. seed oil was able to reduce cellular ROS levels and reduce cell apoptosis. It also shows significant antioxidative effects by decreasing MDA, PC, and 8-OHdG oxidative marker levels and increasing antioxidant enzyme activity. Moreover, LP combination increased the level of nuclear Nrf2, as well as its downstream antioxidant target gene (*HO-1*, *SOD-1*, *MnSOD*, *CAT*, *GPX-1*, and *GPX-4*), and downregulated the mRNA expression levels of the Keap1. All these results show that LP combination proposed a protective effect against H_2_O_2_-induced oxidative damage in HepG2 cells and it may improve cellular oxidative stress by activating the Keap1–Nrf2–ARE signaling pathway.

Based on our results, we defined LP combination as the optimal condition to harvest seed oils in order to ensure a better retention of the antioxidant components. It has great potential in the food industry due to their antioxidant ability and safety to promote the future nutraceutical development and/or functional food investigation from the seed oils. Also, the ability of LP combination to activate the Keap1/Nrf2/ARE signaling pathway in the HepG2 cells may have relevant therapeutic implications.

## Figures and Tables

**Table 1:** Experimental factors and their coded levels.

Factor	Unit	Coded Factor	Range				
			−2	−1	0	1	2
α-T	μM	A	10	20	30	40	50
β- S	μM	B	20	40	60	80	100
SA	μM	C	5	35	65	95	125
CA	μM	D	5	35	65	95	125

**Table 2:** The experimental grouping of HepG2 cells.

Group	Drug	H_2_O_2_ (200 μM)
H_2_O_2_ + G	α-T (10 μM) + β-S (20 μM) + SA (125 μM) + CA (125 μM)	+ +
H_2_O_2_ + T	tea polyphenols (7.11 μM)	+ +
H_2_O_2_ + M	magnolol (7.51 μM)	+ +
Control	—	—
H_2_O_2_	—	+ +

G, group of LP; T, tea polyphenols; M, magnolol; —, not treated by drug/H_2_O_2_; + +, treated by drug/H_2_O_2_.

**Table 3:** Primer sequences used for real-time quantitative PCR.

Gene	Forward Primer	Reverse Primer
*β-Actin*	5′-CAAAGTCTTCCGTGTCCGGG-3′	5′-TCCCTTCCCCTTCCCTGATT-3′
*GPX1*	5′-CAACTTCAGCGCTCTTCGAG-3′	5′-GCGGTGGCATTGTAAGTTGG-3′
*GPX4*	5′-CTTTGCCGCCTACTGAAGCC-3′	5′-CCGAACTGGTTACACGGGAA-3′
*HO-1*	5′-AGGGAATTCTCTTGGCTGGC-3′	5′-GCTGCCACATTAGGGTGTCT-3′
*CAT*	5′-AGTGATCGGGGGATTCCAGA-3′	5′-GAGGGGTACTTTCCTGTGGC-3′
*Keap1*	5′-ACGCGCAGCGATGGAG-3′	5′-CCAGGGTGTAGCTGAAGGTG-3′
*MnSOD*	5′-ATACGCCCCTCTCCTACACA-3′	5′-CGCGGACCATCATAGGTGAG-3′
*SOD1*	5′-GGCTCACACCTCACGTTACA-3′	5′-TGCCATTGAGATTGCCCGAT-3′
*Nrf2*	5′-CTTCTAGTTCGGACGCGGTG-3′	5′-TCAAATCCATGTCCTGTCCCT-3′

**Table 4:** Coded values of the variables used in the CCD design matrix and the corresponding experimental results.

Group	Point Type	A	B	C	D	CAA (μmol QE/100 g)	Group	Point Type	A	B	C	D	CAA (μmol QE/100 g)	Group	Point Type	A	B	C	D	CAA (μmol QE/100 g)
1	Factorial	−1	−1	−1	−1	2.025	12	Factorial	1	1	−1	1	3.733	23	Axial	0	0	0	−2	2.161
2	Factorial	1	−1	−1	−1	4.135	13	Factorial	−1	−1	1	1	6.500	24	Axial	0	0	0	2	4.543
3	Factorial	−1	1	−1	−1	3.567	14	Factorial	1	−1	1	1	6.322	25	Central	0	0	0	0	4.244
4	Factorial	1	1	−1	−1	5.274	15	Factorial	−1	1	1	1	4.622	26	Central	0	0	0	0	4.135
5	Factorial	−1	−1	1	−1	3.131	16	Factorial	1	1	1	1	2.988	27	Central	0	0	0	0	3.990
6	Factorial	1	−1	1	−1	3.024	17	Axial	−2	0	0	0	3.788	28	Central	0	0	0	0	4.646
7	Factorial	−1	1	1	−1	2.970	18	Axial	2	0	0	0	4.791	29	Central	0	0	0	0	4.471
8	Factorial	1	1	1	−1	2.948	19	Axial	0	−2	0	0	4.624	30	Central	0	0	0	0	4.700
9	Factorial	−1	−1	−1	1	4.786	20	Axial	0	2	0	0	3.671	31	Central	0	0	0	0	4.471
10	Factorial	1	−1	−1	1	4.988	21	Axial	0	0	−2	0	4.045							
11	Factorial	−1	1	−1	1	3.860	22	Axial	0	0	2	0	4.187							

A, α-T; B, β-S; C, SA; D, CA.

**Table 5:** CCD model and ANOVA analysis.

Source	Sequential Model Sum of Squares							ANOVA Analysis					
	Sum of Squares	*DF*	Mean Square	F Value	*p*-Value Prob > F	Outcome	Source	Sum of Squares	*DF*	Mean Square	F Value	*p*-Value Prob > F	
Mean vs. Total	523.32	1	523.32				Model	29.12	14	2.08	27.8	<0.0001	significant
Linear vs. Mean	12.61	4	3.15	4.63	0.0059		A	0.65	1	0.65	8.72	0.0094	
2FI vs. Linear	14.75	6	2.46	16.64	<0.0001		B	1.96	1	1.96	26.17	0.0001	
Quadratic vs. 2FI	1.76	4	0.44	5.87	0.0042	Suggested	C	7.39 × 10^−3^	1	7.39 × 10^−3^	0.099	0.7574	
Cubic vs. Quadratic	0.57	8	0.072	0.92	0.543	Aliased	D	10	1	10	133.61	<0.0001	
Residual	0.62	8	0.078				AB	0.28	1	0.28	3.69	0.0726	
Total	553.63	31	17.86				AC	2.13	1	2.13	28.42	<0.0001	
							AD	1.84	1	1.84	24.59	0.0001	
Source	Lack of Fit Tests						BC	2.21	1	2.21	29.57	<0.0001	
Linear	17.27	20	0.86	12.16	0.0026		BD	6.05	1	6.05	80.84	<0.0001	
2FI	2.53	14	0.18	2.54	0.1289		CD	2.24	1	2.24	30	<0.0001	
Quadratic	0.77	10	0.077	1.08	0.4819	Suggested	A^2	3.15 × 10^−3^	1	3.15 × 10^−3^	0.042	0.8399	
Cubic	0.2	2	0.098	1.38	0.3219	Aliased	B^2	0.061	1	0.061	0.81	0.3818	
Pure Error	0.43	6	0.071				C^2	0.083	1	0.083	1.11	0.3078	
							D^2	1.71	1	1.71	22.92	0.0002	
Model Summary Statistics							Residual	1.2	16	0.075			
Source	Std. Dev.	R^2^	Adjusted R^2^	Predicted R^2^	PRESS	Outcome							
Linear	0.83	0.4161	0.3263	0.1056	27.12		Mean			4.11			
2FI	0.38	0.9025	0.8538	0.7743	6.84		C.V.%			6.66			
Quadratic	0.27	0.9605	0.926	0.8344	5.02	Suggested	Adeq Precision			5.02			
Cubic	0.28	0.9795	0.923	0.0512	28.76	Aliased							

A, α-T; B, β-S; C, SA; D, CA.

## Data Availability

The original contributions presented in the study are included in the article, further inquiries can be directed to the corresponding author.

## Abbreviations

α-T	α-Tocopherol
β-S	β-Sitosterol
CA	Canolol
SA	Sinapic acid
LP	Lipid phytochemicals
CCD	Central composite design
CAA	Cellular antioxidant activity
QE	Quercetin
ROS	Reactive oxygen species
MDA	Malonaldehyde
PC	Protein carbonyl
8-OHdG	8-hydroxydeoxyguanosine
SOD	Superoxide dismutase
CAT	Catalase
GPX	Glutathione peroxidase
HO-1	Heme oxygenase-1
T-AOC	Total antioxidant capacity
GSH	Glutathione
Nrf2	Nuclear factor- erythroid 2 related factor 2
Keap1	Kelch-like ECH-associated protein 1
ARE	Antioxidant-response element
